# Non-canonical nucleosides and chemistry of the emergence of life

**DOI:** 10.1038/s41467-018-07222-w

**Published:** 2018-12-12

**Authors:** Sidney Becker, Christina Schneider, Antony Crisp, Thomas Carell

**Affiliations:** Department of Chemistry at the Ludwig-Maximilians Universität München, Butenandtstr. 5-13, 81377 Munich, Germany

## Abstract

Prebiotic chemistry, driven by changing environmental parameters provides canonical and a multitude of non-canonical nucleosides. This suggests that Watson-Crick base pairs were selected from a diverse pool of nucleosides in a pre-Darwinian chemical evolution process.

## Life and LUCA

Life is a highly diverse phenomenon that occupies all conceivable geological niches on Earth. Its development is explained by Darwinian evolution, which must have begun with rudimentary “living” vesicles that at some point transitioned into what we call the last universal common ancestor (LUCA)^1^. LUCA is a hypothetical life form obtained from phylogenetic analysis from which all three kingdoms of life originated^2^. To our understanding, LUCA already possessed the capacity to synthesize specific building blocks such as amino acids, nucleotides and lipids^1^. While phylogenetics allow us to ascertain such information, any events that had occurred prior to LUCA’s emergence remain in the dark, leaving us with only the possibility of simulating plausible prebiotic scenarios in the laboratory.

**Fig. 1:**
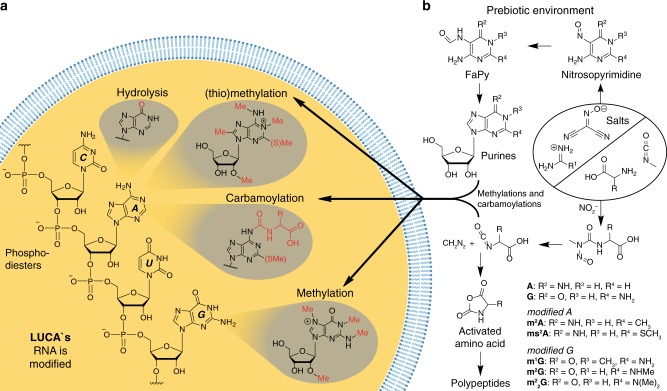
LUCA’s modified bases. **a** The chemical structure of ribonucleic acid (RNA) and of some modified bases. Different (thio)methylation sites and amino acid purine modifications in LUCA are marked in red. **b** Amidine salts are converted into nitrosopyrimidines, which then form formamidopyrimidines. Reaction with ribose provides a set of canonical and non-canonical nucleosides that are assumed to have been present in LUCA

Owing to the discovery of catalytic RNA, it is conceivable that life on Earth emerged from self-replicating RNA oligomers. A prerequisite for this RNA-world concept^3^ is that RNA was present on the early Earth. RNA, however, is a complex molecule (Fig. 1a) that consists of a sugar (ribose), heterocycles (A, C, G and U) for base pairing, and phosphodiesters to link the units. Since the formation of the ribose- and heterocyclic-portions of RNA required different chemistry, we must assume that the early Earth provided areas with different geological conditions to facilitate their syntheses. We can imagine dry desert-type mineralic surfaces that were only occasionally dampened by rain. These mineral fields may have experienced large temperature differences during day and night. Hot fields, close to active volcanos certainly existed with temperatures of above 200 °C, occasionally cooled by rain. We can imagine that much wetter climates existed as well, in which water could dissolve minerals that would later be (re)-precipitated in draughts. pH values may also have varied greatly. Acidic rain generated by NO_x_ and SO_x_ could have given rise to ponds with pH values below 3, while ponds filled with amines and amidines could have had pH values of above 10 or even 12. It seems plausible that the different RNA precursors formed separately in different geological settings and that they were incidentally washed together due to flooding or similar phenomena.

## Non-canonical bases and peptide-RNA hybrids

The chemistry of the early Earth must have been primitive and unspecific. It is hard to imagine that parameters such as temperatures or concentrations of reactants were so tightly controlled that single products formed selectively. Reactions likely took place in vessels such as aqueous ponds or on hot surfaces containing complex mixtures of different minerals. This leads us to believe that the conditions led to the formation of diverse pools of molecules (Fig. 1). Indeed, even today RNA is tremendously structurally diverse. Besides to the four canonical nucleobases (A, C, G and U) RNA contains a phlethora of additional modified nucleosides^4^. Some of these bases are found in all three kingdoms of life, and as such, can be considered to be molecular fossils of the original chemically diverse primordial soup. These non-canonical nucleosides are potential chemical ancestors; relics of early Earth’s chemistry^1^.

### Prebiotic routes to purines and amino acid modified purines

Prebiotic chemical processes likely occurred under conditions far away from defined laboratory chemistry. Today, Chemists use special glassware for their reactions and reactants are added step-wise in a tightly controlled manner, often in a protecting atmosphere and regularly in inert solvents. Concentration and temperature are controlled, and importantly, the reaction product is carefully isolated and purified before it is introduced into the next reaction. All of this was impossible on the early Earth. Prebiotic chemistry forces chemists therefore to think about robust reactions that proceed under “dirty” conditions. Reactions that are general enough that they tolerate different temperatures and concentrations, and that are selective enough to proceed even in mixtures are privileged in a prebiotic setting. They are driven by fluctuations of outside physico-chemical parameters such as day–night or seasonal cycles, which provide intermittent wet and dry conditions along with changing temperatures. Drying out could have triggered selective precipitation and crystallization, which leads to purification and concentration to enable successive reactions^5^. Prebiotic reactions have to work in water, in dry-state conditions, or otherwise in higher-boiling solvents such as formamide. Reductions and oxidations may have occurred in the presence of iron- and sulphur-containing compounds, for example, by the conversion of iron sulphide (FeS) to pyrite (FeS_2_)^6^. High-energy irradiation could also have initiated chemical reactions, particularly when we assume the absence of a shielding ozone layer. Since DNA and RNA are not stable under UV irradiation (*λ* < 300 nm), photocatalysis was probably more important for the formation of small reactive organic molecules. Without sophisticated DNA/RNA repair mechanisms, oligonucleotides could survive only in niches devoid of UV light^7^. It is safe to assume that UV light was a thread to early life.

**Fig. 2:**
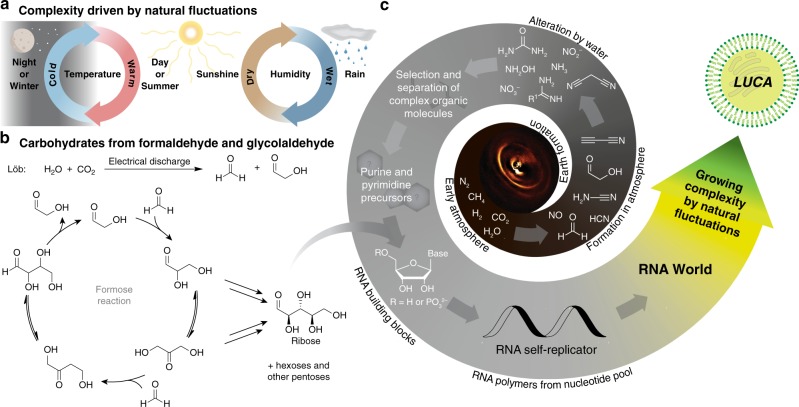
Natural fluctuations steer chemical transformations towards greater complexity. **a** Molecular complexity driven by fluctuations of physico-chemical conditions, such as day–night or seasonal changes, leading to wet–dry cycles. **b** Since the early Earth was not uniform, carbohydrates could have emerged separately from nucleobases or their precursors e.g. via the formose reaction. **c** When washed into the same environment, different nucleosides/nucleotides could have formed

When we think about the formation of small prebiotic starting molecules, electrical discharge needs to be considered (Fig. 2a). In a nearly neutral N_2_ atmosphere composed also of H_2_O, CO_2_, H_2_ and CH_4_, electrical discharge converts N_2_ into NO^8^ which can be reduced to NH_2_OH and then into NH_3_. In addition, N_2_ reacts with CH_4_ under discharge conditions to give products including HCN, cyanamide and cyanoacetylene^9^. Under such electrical discharge conditions (Fig. 2a) humid CO_2_ produces the starting materials for sugars such as formaldehyde and glycolaldehyde (Fig. 2b). This all together provides a rather large set of small prebiotic organic molecules that can act as starting materials for the formation or RNA nucleosides (Figs. 1b and  2b).

Cyanamide, for example, can give different amidinium compounds by nucleophilic addition, which form low-melting organic salts with nitrosated malononitrile (Fig. 1b)^5^. We found that that these salts form formamidopyrimidines (FaPys, Fig. 1b), which react efficiently with ribose to give purine nucleosides^10^. This new FaPy-pathway generates not only the two canonical purine bases (A and G), but also a variety of RNA modifications^5^. Under special conditions, even amino acid modified purine nucleosides are generated. Most importantly all these modified purine bases are found today in contemporary RNA, thus strengthening the idea that non-canonical RNA bases are vestiges of our primordial anscestor^11^.

### Ribose and the oligomerization problem

The use of ribose as a prebiotic starting material for nucleoside formation is sometimes criticised because no clear prebiotically plausible route to ribose has been found. We still believe that ribose was present on the early Earth and that we have simply to discover the right conditions. It is well known that formaldehyde and glycolaldehyde produce ribose in the formose reaction (Fig. 2b), but admittedly, the yields are low (<1%)^12^. It is known that borates increase the yield of ribose^13^ and we are sure that with more time and research even better and more selective conditions will be found. We should also not forget that life could have begun with oligonucleotides composed of sugars other than ribose^14^. Even ribose can exist in a 5- or a 6-membered ring form. The 5-membered arrangement (furanosides) are what today constitute our RNA, but we know from A. Eschenmosers’ seminal work that the 6-membered pyranosidic RNA also produces wonderful double strands with selective base pairing^15^. It is a riddle, why the thermodynamically less favoured 5-membered furanosides were chosen to create life. One argument is that the 5-membered furanosides are more easily phosphorylated because they possess a very reactive primary hydroxyl group^16^. Such phosphorylation is needed to stitch the nucleosides together to enable strand formation^17^. Given that prebiotic RNA nucleosides and consequently RNA strands may have been structurally diverse, containing for example, amino acid modifications, we can envision that some had physico-chemical properties or catalytic properties that offered a survival advantage. Pre-Darwinian evolution may have acted first on molecules rather than living species to select the fittest, initially maybe just the most stable molecules or RNA strands (Fig. 2c). At some point nature must have discovered that the ultimate solution to molecular survival is reproduction via self-replication and catalysis. This is best achieved in a shielded environment within which the molecules needed for replication can be autonomously generated (metabolism). This unit is called a cell.
