# Publisher Correction: Non-canonical nucleosides and chemistry of the emergence of life

**DOI:** 10.1038/s41467-019-08340-9

**Published:** 2019-01-15

**Authors:** Sidney Becker, Christina Schneider, Antony Crisp, Thomas Carell

**Affiliations:** Department of Chemistry at the Ludwig-Maximilians Universität München, Butenandtstr. 5-13, 81377 Munich, Germany

Correction to: *Nature Communications*; 10.1038/s41467-018-07222-w; published online 12 December 2018

The original version of this Article contained errors in the citations in the second, third and fourth sentences of the first paragraph of the ‘Life and LUCA’ section, which incorrectly read ‘Its development is explained by Darwinian evolution, which must have begun with rudimentary “living” vesicles that at some point transitioned into what we call the last universal common ancestor (LUCA)^2^. LUCA is a hypothetical life form obtained from phylogenetic analysis from which all three kingdoms of life originated^3^. To our understanding, LUCA already possessed the capacity to synthesize specific building blocks such as amino acids, nucleotides and lipids^2^.’ The correct version states ‘(LUCA)^1^’ in place of ‘(LUCA)^2^’, ‘originated^2^’ instead of ‘originated^3^’ and ‘lipids^1^’ rather than ‘lipids^2^’. This has been corrected in both the PDF and HTML versions of the Article.

